# Catalytic Activity of Defect-Engineered Transition Me tal Dichalcogenides Mapped with Atomic-Scale Precision by Electrochemical Scanning Tunneling Microscopy

**DOI:** 10.1021/acsenergylett.2c02599

**Published:** 2023-01-16

**Authors:** Marco Lunardon, Tomasz Kosmala, Mahdi Ghorbani-Asl, Arkady V. Krasheninnikov, Sadhu Kolekar, Christian Durante, Matthias Batzill, Stefano Agnoli, Gaetano Granozzi

**Affiliations:** † Department of Chemical Sciences, University of Padova, Padova 35131, Italy; ‡ Institute of Experimental Physics, University of Wrocław, Wrocław 50-204, Poland; § Institute of Ion Beam Physics and Materials Research, Helmholtz-Zentrum Dresden-Rossendorf Dresden 01328, Germany; ∥ Department of Applied Physics, Aalto University, 00076 Aalto, Finland; ⊥ Department of Physics, University of South Florida, Tampa, Florida 33620, United States; # INSTM Research Unit, University of Padova, Padova 35131, Italy

## Abstract

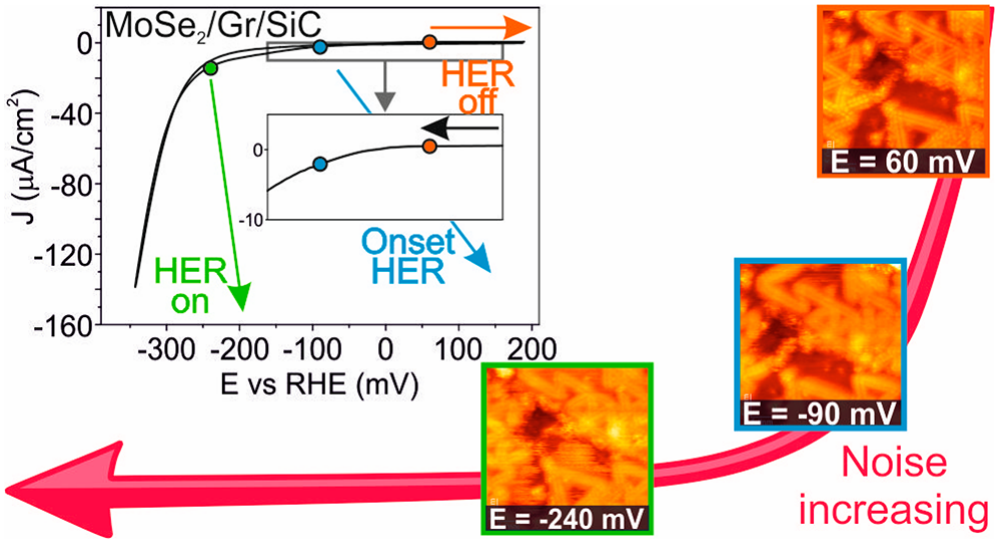

Unraveling structure–activity relationships is a key objective of catalysis. Unfortunately, the intrinsic complexity and structural heterogeneity of materials stand in the way of this goal, mainly because the activity measurements are area-averaged and therefore contain information coming from different surface sites. This limitation can be surpassed by the analysis of the noise in the current of electrochemical scanning tunneling microscopy (EC-STM). Herein, we apply this strategy to investigate the catalytic activity toward the hydrogen evolution reaction of monolayer films of MoSe_2_. Thanks to atomically resolved potentiodynamic experiments, we can evaluate individually the catalytic activity of the MoSe_2_ basal plane, selenium vacancies, and different point defects produced by the intersections of metallic twin boundaries. The activity trend deduced by EC-STM is independently confirmed by density functional theory calculations, which also indicate that, on the metallic twin boundary crossings, the hydrogen adsorption energy is almost thermoneutral. The micro- and macroscopic measurements are combined to extract the turnover frequency of different sites, obtaining for the most active ones a value of 30 s^–1^ at −136 mV vs RHE.

The production of *green* hydrogen by water electrolysis is a key step to establish a sustainable energy infrastructure.^1^ However, the development of electrochemical (EC) water-to-hydrogen conversion devices through the hydrogen evolution reaction (HER) is limited by the materials science and engineering aspects of the working electrodes.^2−4^

Nowadays, transition metal dichalcogenides (TMDs) are investigated as replacements for Pt group materials^5^ (PGMs), in the hope that they can achieve similar performances but at a lower cost.^6−9^ These materials have drawn the scientific community’s attention due to their diverse electronic,^10−13^ optical,^13−16^ magnetic,^17,18^ and catalytic^19−21^ properties that can be easily tuned through chemical composition,^22,23^ crystal phase,^24,25^ and thickness.^10^ Moreover, the TMD properties can be further improved by defects^26,27^ or strain,^28^ which can be introduced during or after the synthesis. Regarding the HER, the EC performances of standard TMDs are inferior to those of PGM-based catalysts due to their limited number of active sites and catalytically inert basal plane. However, many efforts have been made to develop strategies for minimizing the performance gap between these two classes of materials.^29,30^

Previously, we investigated an uncharted strategy for improving the HER activity of TMDs by exploiting a particular type of line defects referred to as mirror twin boundaries (MTBs),^21,31^ i.e., the boundary separating two grains rotated by 60°. Contrary to serpentine or low-angle grain boundaries,^32,33^ which are formed by the coalescence of arbitrarily orientated crystal domains, the formation of MTBs becomes energetically favorable in non-stoichiometric chalcogen-deficient TMDs,^34^ and can be promoted through post-synthesis strategies.^35,36^ The presence of MTBs implies atoms with modified coordination that induce different electronic properties, and spectroscopic investigations suggest that MTBs have metallic properties.^21,37^ By analogy with the different performance exhibited by the semiconducting 2H vs 1T metallic TMD polymorphs,^25^ MTBs are also expected to have high catalytic activity.^21^

Thence, defect-engineering in TMDs can have a huge impact on the EC activity.^38−40^ However, an accurate correlation between an atomically well-defined site and its catalytic activity is challenging because standard EC characterization techniques provide only area-averaged information, so connecting specific figures of merit to a single type of microscopic defect is not trivial, given that various structural and morphological defects can be co-present on the same “real” catalyst. To surpass these limitations, we developed a novel method to study electrocatalytic sites with atomic resolution in *operando* conditions by evaluating the noise in electrochemical scanning tunneling microscopy (EC-STM), which allowed us to investigate separately a wide gamut of defects at the atomic scale.^41,42^

Here we present a structural and EC investigation of a MoSe_2_ monolayer (ML) grown by molecular beam epitaxy (MBE) on a bilayer of graphene (Gr) supported on a 6H-SiC(0001) single crystal (MoSe_2_/Gr/SiC). Such MBE-grown MoSe_2_ films exhibit a high density of MTBs. On this system, given the high spatial resolution of EC-STM, we could investigate the activity not only of linear MTBs, but also of the point defects created locally by the intersections of MTBs.

## Structural Characterization

Before the EC-STM measurements, the chemical composition of the MoSe_2_/Gr/SiC sample was determined by X-ray photoelectron spectroscopy (XPS). The survey spectrum of the catalyst (see Figure S1a) shows peaks due to Si 2p, C 1s, Mo 3d, and Se 3d, besides the signal due to O 1s (due to air exposure). Figure S1b shows the high-resolution photoemission spectra of Mo 3d and Se 3d and their deconvolution into chemically shifted components. The Mo 3d region can be separated in three doublets and an additional single peak at a binding energy (BE) of about 229 eV, corresponding to the Se 3s levels. The main doublet, with the Mo 3d_5/2_ and 3d_3/2_ peaks centered at 228.7 and 231.8 eV, respectively, is attributed to Mo(IV) species of 2H-MoSe_2_ (80.7 at.%).^21,43,44^ The other two, less intense doublets can be associated with Mo(V) species (10.2 at.% with Mo 3d_5/2_ at 230.5 eV) due to substoichiometric MoO_3-*x*_ or complex structural configurations such as Se–Mo–O bonds^45,46^ and Mo(VI) species (9.1 at.% with Mo 3d_5/2_ at 232.4 eV) deriving from surface oxidation (e.g., MoO_3_).^43,44,47^ The Se 3d core level is made up by a single doublet with peaks at 54.2 and 55.1 eV, which corresponds to Se 3d_5/2_ and Se 3d_3/2_ levels, indicating the presence of Se^2–^ ions in 2H-MoSe_2_.^21,43,44^ Additional XPS analysis was performed immediately after the EC-STM measurements to evaluate the changes induced by the potentiodynamic experiment (see Figure S1b and Table S1). Both the Mo(V) and Mo(VI) components decreased, as expected for the oxide phases in an acid solution under reducing working conditions. XPS analysis of the O 1s region provides direct evidence of the Mo-oxide reduction as suggested by the decrease of the photoemission signal intensity in the BE region from 529.9 to 530.9 eV, which can be associated with Mo-oxides (see Figure S1C).^47−49^

**Figure 1:**
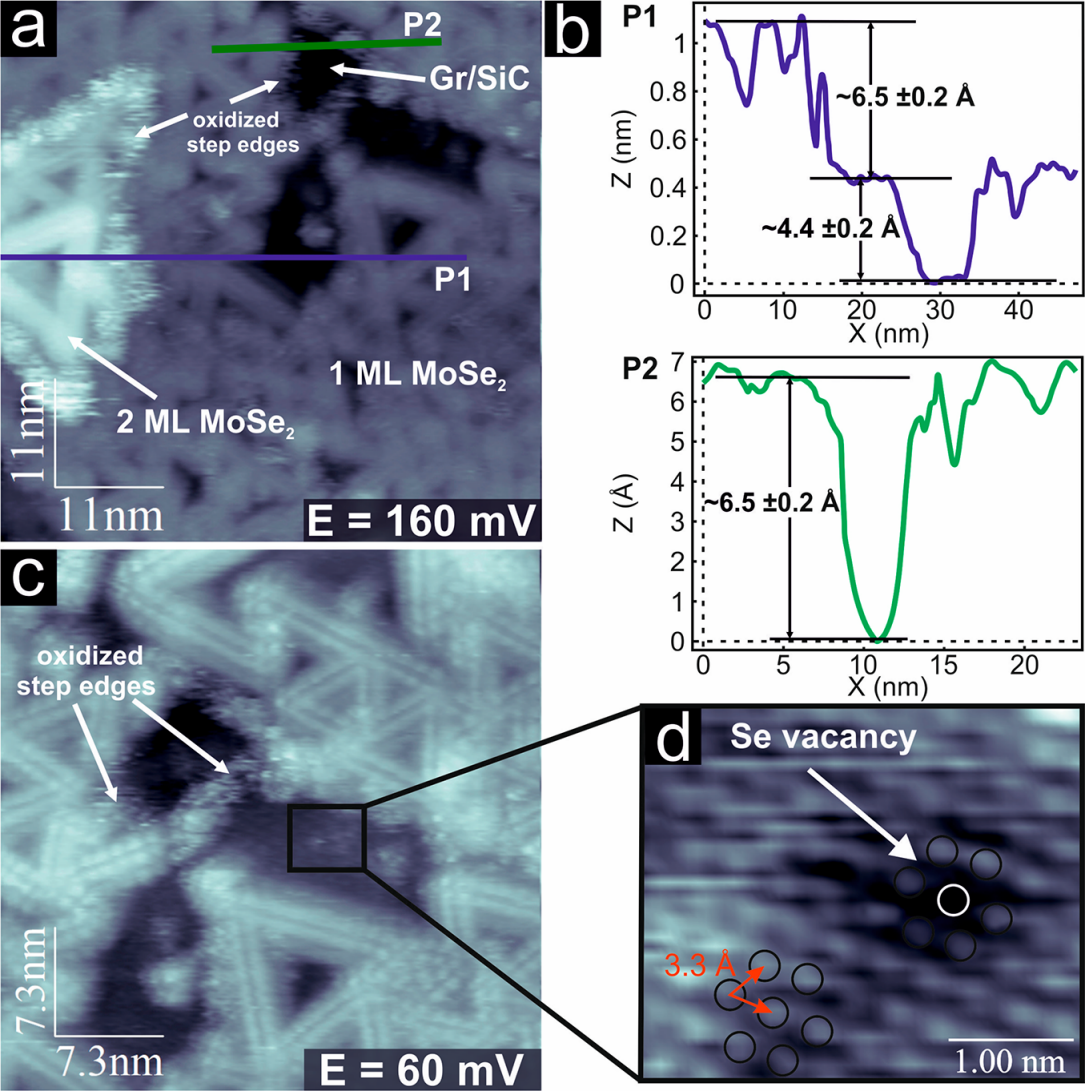
(a) Large-scale *in situ* EC-STM image of MoSe_2_/Gr/SiC acquired at 160 mV vs RHE electrode potential, *I*_T_ = 1.84 nA, *U*_B_ = 89 mV. (b) Height profiles of the P1 and P2 lines in panel a. (c) Large-scale *in situ* EC-STM image of MoSe_2_/Gr/SiC acquired at 60 mV vs RHE electrode potential, *I*_T_ = 1.84 nA, *U*_B_ = 89 mV. (d) Atomically resolved *in situ* EC-STM image of the basal plane of MoSe_2_ at 60 mV vs RHE, *I*_T_ = 1.84 nA, *U*_B_ = 89 mV.

The same sample was further investigated by EC-STM in Ar-saturated electrolyte (0.1 M HClO_4_). Figure 1a and c shows some large-scale *in situ* EC-STM images of MoSe_2_/Gr/SiC recorded in precatalytic conditions. Most of the support is covered by a single layer of MoSe_2_; however, some uncovered Gr areas and double-layer MoSe_2_ islands can be observed, too. The MoSe_2_ film shows highly corrugated straight lines that cross each other, forming a triangular pattern. As shown in Figure S2, the lengths of the triangle’s sides are mainly 7.8 ± 0.4 nm (65%) or 4.5 ± 0.4 nm (35%), while larger features are rarely observed. The line defects appear as a pair of parallel atomic rows, which can be assigned to the position of the Se-atoms in the MTB in agreement with previous works.^21,37,50−53^

The topographic profiles measured along the P1 and P2 lines in Figure 1a are shown in Figure 1b and indicate a layer height of 6.5 ± 0.2 Å, consistent with the three-atomic-layer (Se-Mo-Se) ML thickness, suggesting a first and second layer of MoSe_2_ on Gr/SiC substrate. The step edges, regardless of the applied electrode potential, exhibit a fuzzy contrast, which can be attributed to the oxidized phase previously detected by XPS. Only occasionally are clean step edges with a sharp profile observed (Figure S3). This suggests that step edges are more prone to oxidation compared to rest of the basal plane due to their metallic nature and the presence of unsaturated bonds.^37,54^ Moreover, an apparent height of 4.4 ± 0.2 Å between MTBs and the basal plane can be identified in the line profile P1, confirming the strong difference in the electronic structures between the two.^51^ The atomically resolved EC-STM image of the basal plane shows the presence of a flat monolayer with a well-ordered hexagonal lattice with a periodicity of 3.3 ± 0.1 Å, occasionally showing point defects like the dark spot marked by a white circle in Figure 1d. This point defect is centered on a site normally occupied by Se anions, which by comparison with other STM works can be associated with a Se-vacancy.^53,55^

It is worth noting that, since the MTBs are clearly observed with atomic resolution by EC-STM, such defects are stable after exposure to air and in the presence of acid electrolyte.

## Electrocatalytic Characterization

To map with atomic resolution the catalytic activity of the MoSe_2_ surface as a function of the applied electrode potential, we performed *operando* potentiodynamic EC-STM in an EC potential window between 160 mV and −340 mV vs RHE at steps of 50 mV.

Figure S4 shows a selection of EC-STM topographic images summarizing the potentiodynamic experiment. No significant changes are observed above −40 mV vs RHE (Figure S4c), while at this potential, some localized spikes become visible near the Se-vacancy site, and increase as the EC potential is decreased. Additional highly corrugated spots appear along the MTBs at −190 mV vs RHE, which eventually propagate also on the basal plane at a more negative potential (Figure S4g–i). Sweeping back the electrode potential toward the positive direction, the noise fluctuations on the basal plane, along MTBs, and at the Se-vacancy are damped (see Figure S4i–p). The full data set of the experiment is presented as a movie, together with a detailed description in the SI.

The activation/suppression of noise with the EC potential is easily explained: when the working electrode potential (*E*) is set to an EC potential where a reaction takes place, the rapid local changes produced by the reaction (formation or disappearance of charged species, adsorption/desorption phenomena) perturb the tip–sample tunneling junction. We have recently proposed evaluating the noise fluctuations during the EC potentiodynamic run by introducing a new quantity, the tunneling current roughness (*cr*),^41,42^ which quantifies the variations of the tunneling current with respect to the value set in the feedback loop of the STM working in constant current mode. The *cr* can be calculated with subnanometer resolution on any point of interest of an EC-STM image. Across an EC potential window where a reaction is activated, the *cr* values versus *E* show a sigmoidal profile that describes the off/on switch of the EC reaction.^41^ By the fit of the sigmoidal curve, it is possible to extract figures of merit such as the onset reaction potential (*E*_on_) and the attenuation factor (*a*) of each site. Herein, for the quantitative analysis we will use the *faradaic-cr* (*L*) values, which we demonstrated to represent the size-normalized noise contribution of the faradaic processes, excluding any noise component due to the intrinsic structural features of the site (i.e., topographic contributions).^41^ More details about this technique and the data analysis are reported in the Supporting Information (SI).

**Figure 2:**
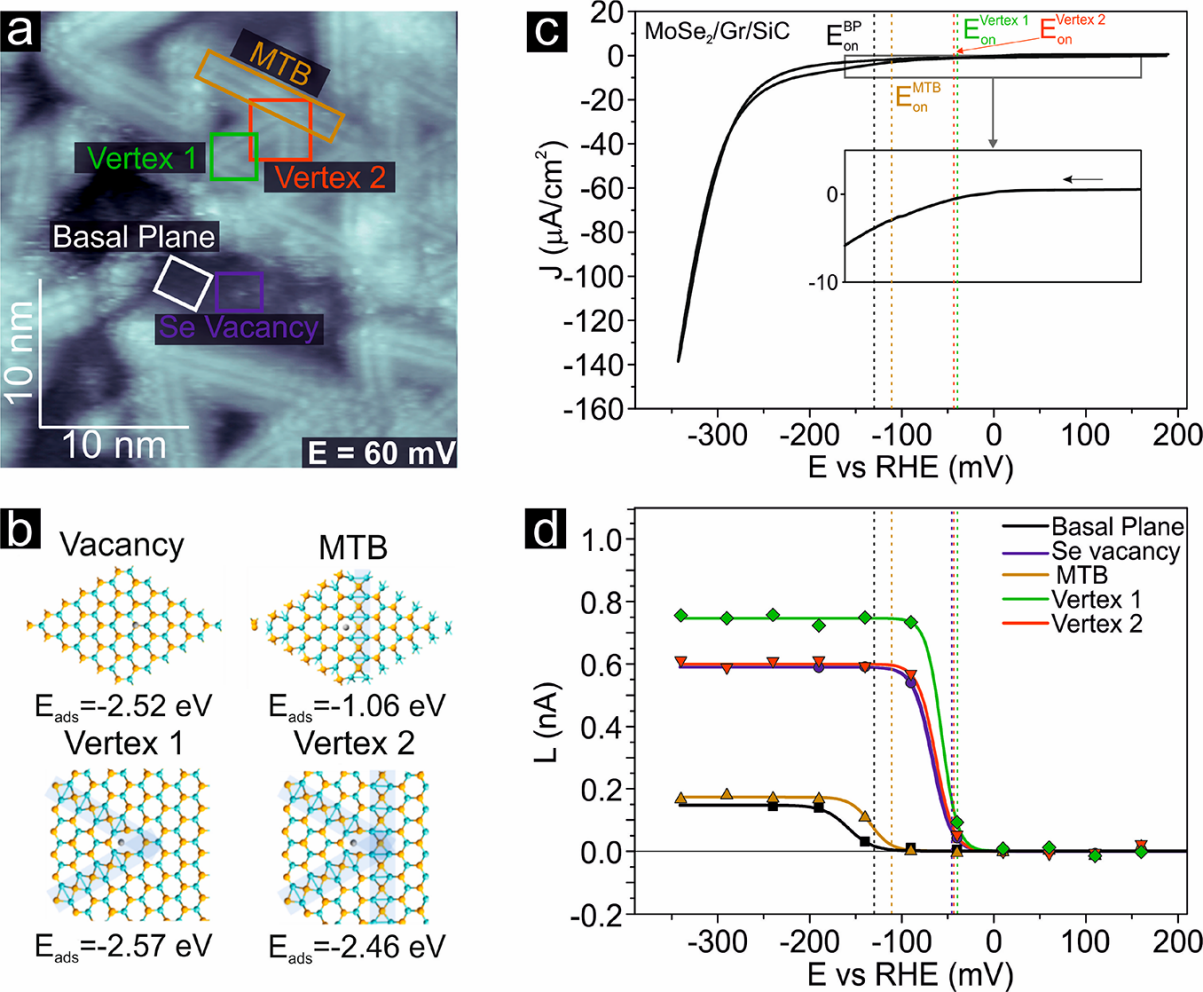
(a) Large-scale *in situ* EC-STM image of MoSe_2_/Gr/SiC acquired at an electrode potential of 60 mV vs RHE, *I*_T_ = 1.84 nA, *U*_B_ = 89 mV. (b) Atomic structures of MoSe_2_ monolayer with adsorbed H-atom on pristine 44 |P MTB and two different vertices. The adsorption energies are also listed. (c) Cyclic voltammogram of the MoSe_2_/Gr/SiC recorded in Ar-saturated 0.1 M HClO_4_, scan rate: 20 mV s^–1^. (d) Fit results of the *faradaic-cr* (*L*) as a function of the EC potential extracted from the highlighted rectangles in panel a.

Figure 2 shows the topographic EC-STM image of the examined area, with colored rectangles and labels identifying different surface structures. The *cr*-analysis has been performed on a basal plane (BP), an MTB, a Se-vacancy, and different types of vertex sites on the MoSe_2_/Gr/SiC surface. Figure 2b shows the ball-and-stick models of the investigated sites for H adsorption, such as two types of vertices (named vertex 1 and vertex 2) which are produced by different intersections of MTBs.

The macroscopic cyclic voltammogram (CV) and the local *L* vs *E* plots are reported in Figure 2c. The different *cr* profiles shown in Figure 2d reflect the different catalytic activity of the various sites.

Different values of *E*_on_ are evident in Figure 2d, with the *E*_on_ of the MTB and vertex sites showing a lower overpotential of 20 and 90 mV, respectively, compared to the *E*_on_ of the BP (−130 mV vs RHE).

**Figure 3:**
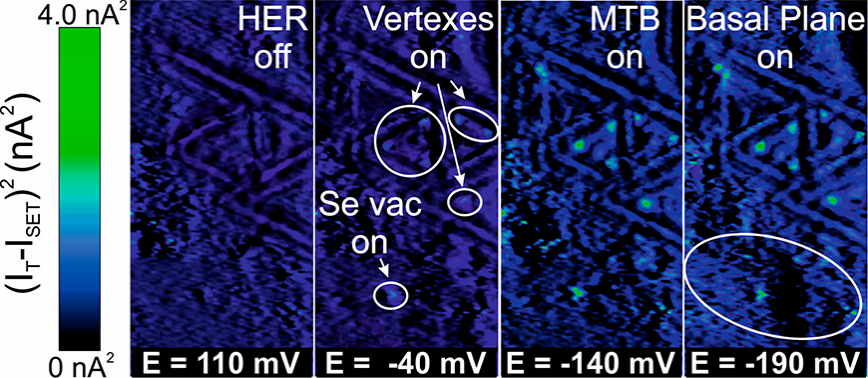
Squared deviation of tunneling current signal relative to the *I*_SET_ of investigated sites in Figure 2a at 110, −40, −140, and −190 mV vs RHE.

To provide a direct map of chemical activity, Figure 3 shows the squared deviation of the tunneling current signal relative to the *I*_SET_ of the investigated area at selected electrochemical potentials, moving from precatalytic to catalytic HER conditions. As discussed in Supporting Note 2, (*I*_T_ – *I*_SET_)^2^ is the key quantity to evaluate the noise and therefore represents the most direct way to visualize the noise hot spots at the different electrochemical potentials. At first, higher values of current roughness are observed at −40 mV vs RHE on the vertex sites, while further noise spots appear also on the MTBs and basal plane sites at −140 and −190 mV vs RHE, respectively.

**Figure 4:**
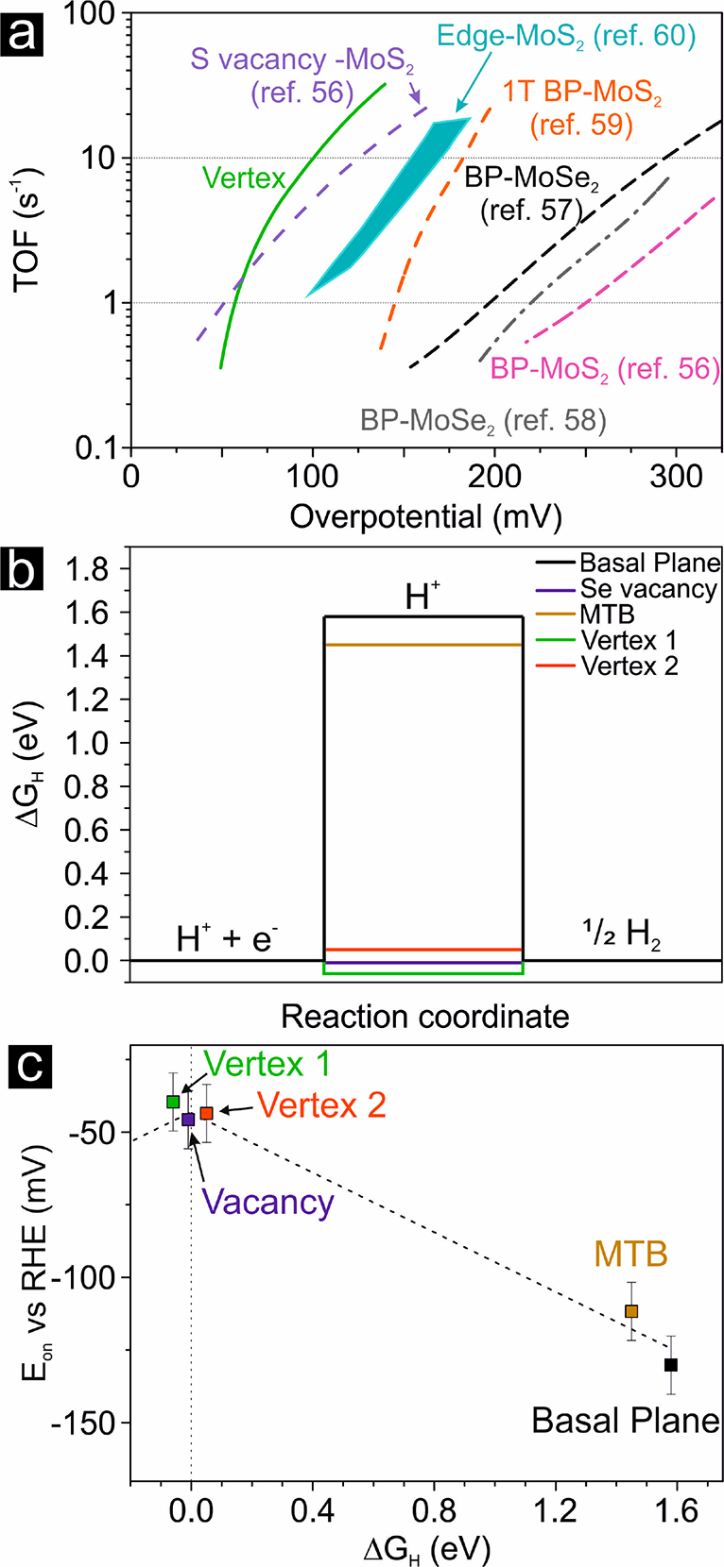
(a) *av*-TOF as a function of the overpotential per the vertex sites based on Figure 2c; literature results per common TMD sites are also shown for comparison (dashed line and shaded area): basal plane-MoS_2_ from ref (56), basal plane-MoSe_2_ from refs (57 and 58), edge-MoS_2_ from ref (60), 1T basal plane-MoS_2_ from ref (59), and S-vacancy-MoS_2_ from ref (56). (b) Gibbs free energy profiles of HER on a pristine MoSe_2_ monolayer and with different types of defects. (c) Plot of onset potential versus Gibbs free energy of HER showing a linear relationship.

The high spatial density and distinct geometry of MTBs produce several active sites arising from their intersections, which significantly contribute to the activity of the planar film. The areal density of active sites, consisting of any type of vertex, estimated from the topographic images in Figures 1a and S2 is approximately (3 ± 0.1) × 10^12^ sites·cm^–2^. Therefore, considering the lower *E*_on_ exhibited by vertices according to local *cr* plots, the macroscopic EC behavior of the MoSe_2_/Gr/SiC at the early onset of HER can be associated with these sites, as highlighted in Figure 2c. Hence an average turnover frequency (*av*-TOF) for the vertex sites can be calculated (see Supporting Note 3). In Figure 4a, the obtained *av*-TOF trend for the vertex sites (TOF = 30 s^–1^ at an overpotential of 136 mV vs RHE) is compared with the literature values^56−60^ to highlight the different intrinsic activity among various TMD sites. The *av*-TOF of vertex sites is higher than that of unmodified MoSe_2_,^57,58^ which is mainly connected to the basal plane activity, and it is even higher than MoS_2_-edges^60^ and 1T-MoS_2_,^59^ which are well-known to be among the most active TMD sites for HER.^4,25,61^ It must be noted that the sigmoidal curves of vertex 2 and Se-vacancy are strongly overlapping, suggesting a very similar catalytic activity for these two sites. The MTBs are thermodynamically stable under chalcogen-deficient conditions;^34,35,48,50^ the MoSe_2_ film can accommodate a systematic deficiency of Se-atoms through a local variation of the stoichiometry and of the metal–chalcogenide coordination.^34^ Isolated Se-vacancies, far from the MTBs, therefore are statistically uncommon defects on the catalyst surface, as confirmed by the present data and previous investigations.^37,50,52^ Hence, the effect of Se-vacancies on the catalytic activity can be considered negligible. Anyway, our results (i.e., low onset potential for HER) confirm previous theoretical and experimental works indicating that chalcogenide vacancies are very active catalytic centers.^62−64^

Additional information can be deduced by analyzing the attenuation factor *a*: as previously discussed,^41^ the different growth rates of the sigmoidal profile can be associated with differences in the local value of the Tafel slope. The growth rate parameters are reported in Figure S5a, and the lower *a* value of the vertex sites compared to that of the basal plane suggests a more favorable hydrogen adsorption step. This conclusion is partially supported by the value of 89 mV/dec of the macroscopic Tafel slope measured in the vertex *E*_on_ region, which is significantly lower than the value reported in the literature for the TMC basal plane sites (120 mV/dec, see Figure S5b).^65,66^ However, it must be emphasized that, due to nonlinearity, the 89 mV/dec local value is only an estimation in a small range near to the onset potential. Therefore, any information about the catalytic activity obtained by the local Tafel slope must be necessarily considered highly speculative.

The comparative analysis of the *L*(*E*) curves indicates that the vertices are the most catalytically active sites, followed by the MTBs and BP. This agrees with the direct visualization of catalytically active sites during an EC reaction, summarized in Figures 3 and S4, and density functional theory (DFT) calculation results (see Figure 4b and c). The different structural units were modeled, and the hydrogen Gibbs free energy of adsorption, Δ*G*_H_, was calculated, since it is considered a reliable descriptor of the HER in acid conditions. Details about the DFT calculations are reported in the SI.

The energetics of hydrogen adsorption on pristine and defective MoSe_2_ were assessed using DFT calculations. We have considered various hydrogen adsorption sites, and the most energetically favorable configurations are shown in Figure 2b. Vertices are created by joining the 44 |P MTBs with 60° relative orientation. We have investigated several vertex configurations since the precise atomic structure and composition of these vertices are unknown. It should be mentioned that the distance between the vertices where the MTBs intersect is noticeably smaller than in the experiment, suggesting a higher concentration of vertices in our simulations.

In the case of a pristine monolayer, the H-atoms prefer to be in the interstitial configuration rather than adatom configurations due to the large primitive cell of MoSe_2_, which provides free space in the hollow site of the Mo-atom plane, as demonstrated earlier.^67,68^ In the presence of MTB, the H-atom is adsorbed at the center of the hexagon next to the MTB. We note that due to metallic nature of the one-dimensional metallic band at the MTB, the adsorption energy of H-atoms in general depends on the occupancy of the band, i.e., the position of the Fermi level. This also gives rise to a difference of about 0.7 eV in the adsorption energy of H-atom when MoSe_2_ with the MTB is modeled as a ribbon^67^ due to the charge transfer between the metallic states localized at the edges and the MTB.^34^ While the MTB does not have any dangling bonds, the vertices contain more complex morphologies with only partially unsaturated bonds, therefore affecting adsorption. It has been demonstrated that several of these vertices serve as preferential nucleation sites for vapor-deposited metal atoms.^69^ Our calculations showed that the adsorption energies of H-atoms on vertices are lower than those on MTBs and Se-vacancies. The strongest interaction between the H and defects was found for vertex 1, with the adsorption energy of −2.57 eV.

To achieve more insight into the role of defects in HER reactivity, we further compared the free energy diagrams for the basal plane, Se-vacancy, MTB, and vertices (Figure 4b). The vertices exhibit the lowest HER energy among the different considered defects. The reported values must be intended as relative indicators of the different catalytic activities of the investigated sites, since they were calculated considering the adsorption of a single H-atom, but it is reported that the *E*_ads_ and Δ*G*_H_ change as a function of the H coverage.^70^ Therefore, further investigations were carried out: different possibilities of adsorption positions have been investigated for vertex 1, considering an increasing number of adsorbed H-atoms. The most stable configurations are reported in Figure S6, and the results show that the interaction of hydrogen with vertices decreases with increasing hydrogen coverage. However, the vertices adsorb H much more strongly than the basal plane and MTBs. The free energy plot indicates that the HER energy barrier of the vertices is still lower than that of other defects, suggesting a high activity of vertices toward the HER process on the MoSe_2_ ML (see Figure S6b).

The sequence of reactivity experimentally observed by *cr*-EC-STM perfectly agrees with the trend of Δ*G*_H_ obtained by the DFT simulations. In close analogy to what was observed in a previous investigation,^42^ the onset potential obtained by the *cr* curves is indeed linearly correlated to the Δ*G*_H_, as shown in Figure 4c.

In this work, we have shed light on a new strategy to activate the HER on the MoSe_2_ basal plane.^41^ We confirmed that metallic defects like MTBs are very stable and moderately active; however, the high resolution capabilities of the *cr* analysis allowed us to identify in secondary structures such as the intersections of MTBs, i.e., the vertices, the most active sites, which are extremely active even at a very low overpotential (TOF = 30 s^–1^ at an overpotential of 136 mV vs RHE), as also confirmed by DFT calculations (Δ*G*_H_ almost perfectly thermoneutral for vertex 1). This work demonstrates the huge potential of EC-STM in the precise identification and quantification of active sites and also proposes new strategies for the activation of the basal plane of TMDs.

## Methods and Materials

Mono- to bilayer MoSe_2_ films were grown by molecular beam epitaxy. Mo was evaporated from a home-built water-cooled mini e-beam evaporator from a solid, 2 mm diameter, high-purity Mo rod. Atomic selenium was supplied by a valved, hot-wall selenium cracker source. The films were grown in more than 10 times higher chalcogen than molybdenum flux at a growth temperature of 300 °C. The growth rate was slow, at about 1 h per monolayer. More details about the growth and vacuum characterization of the samples can be found in ref (37).

The EC-STM measurements were carried out using a home-built electrochemical scanning tunneling microscope at constant current mode, as described by Wilms et al.^71^ The electrolyte was prepared by using deionized water from a Millipore-Pure (Merck, Burlington, MA, USA) water system (with a specific resistance of 18 MΩ·cm and a residual amount of organic impurities in the ppb regime) and purged with supra pure argon gas for several hours before use. During the EC-STM measurement, the chamber was filled with Ar gas. Chemicals used (purchased from Sigma-Aldrich, Saint Louis, MO, USA) were of the highest commercially available quality and were used without further purification. The tunneling tips were electrochemically etched from a 0.25 mm tungsten wire in 2 M KOH solution and subsequently cleaned in high-purity water, dried, and coated by passing the tip through a drop of hot polymer glue (Pattex, Germany) placed on a platinum sheet with a 0.5 mm hole. The usual temperature of the glue is 150 °C. Each fabricated tip is tested directly in the EC-STM cell to verify the faradaic current leakage before use. The test is performed by changing the bias of the tip with respect to the working electrode in the range ±500 mV. If the leakage exceeds 50 pA, the tip is unsuitable for measurements and discarded. More details on the tip quality can be found in Supporting Note 4 and Figure S7. Platinum wires were used as counter and reference electrodes to ensure the system’s high purity; a correction factor of 0.8 V was considered for conversion from the Pt/PtO reference electrode to RHE.^72^ The stability of the Pt wires reference electrode was checked by comparing the results of the *faradaic-cr* analysis with a further cycle of potentiodynamic STM images on the same area, as reported in Figure S8.

The image analysis was carried out by using the WSxM 5.0 software.^73^

The XPS analysis was carried out using an EA 125 Omicron electron analyzer equipped with five channeltrons, working at a base pressure of 2 × 10^–10^ mbar. The XPS data were collected at RT with the Mg Kα line (*h*ν = 1256.6 eV) of a non-monochromatized dual-anode DAR400 X-ray source using 0.1 eV energy steps, 0.5 s collection time, and 20 eV pass energy. The binding energy (BE) scale was calibrated using a gold sample (Au 4f at 84 eV). Curve-fitting of Mo 3d and Se 3d lines was performed employing a Voigt function with a Shirley background using the KolXPD software.

Details about the DFT calculations are reported in Supporting Note 5.

## Supporting Information Available

Figures S1–S8 and Table S1, showing X-ray photoemission spectra (survey and core level spectra) of the sample before and after electrochemistry; additional STM topographies; wider data set about the potentiodynamic experiment both as series of EC STM images as a function of the electrochemical potential and as a movie (described in detail in Supporting Note 1); details about the analysis of the noise in the tunneling current (Supporting Note 2), TOF measurements (Supporting Note 3), the EC-STM tip (Supporting Note 4), and DFT calculations (Supporting Note 5) (PDF)
Movie of the *in situ* potentiodynamic EC-STM experiment showing a direct correlation of the changes observed on the surface by the STM as a function of the applied electrode potential in anodic and cathodic directions to the cyclic voltammetry curve (MP4)
