# Supplementary material for: Catalytic Activity of Defect-Engineered Transition Me tal Dichalcogenides Mapped with Atomic-Scale Precision by Electrochemical Scanning Tunneling Microscopy
Source: ACS Energy Lett. 2023 Jan 16;8(2):972–80. doi: 10.1021/acsenergylett.2c02599 (PMC9926491; doi:10.1021/acsenergylett.2c02599)
Supplement: Supplementary file 1 — nz2c02599_si_002.pdf [file nz2c02599_si_002.pdf]

Supporting Information

Catalytic Activity of Defect-Engineered Transition Metal Dichalcogenides Mapped with Atomic Scale Precision by Electrochemical Scanning Tunneling Microscopy

Marco Lunardon[1], *Tomasz Kosmala*[1,2*], *Mahdi Ghorbani-Asl*[3], *Arkady V. Krasheninnikov*[3,4], *Sadhu Kolekar*[5], *Christian Durante*[1], *Matthias Batzill*[5], *Stefano Agnoli*[1,6]* *and Gaetano Granozzi*,[1,6]

[1]Department of Chemical Sciences, University of Padova, Padova 35131, Italy

[2]Institute of Experimental Physics, University of Wrocław, Wrocław 50-204, Poland

[3]Helmholtz-Zentrum Dresden-Rossendorf Institute of Ion Beam Physics and Materials Research Dresden 01328, Germany

[4]Department of Applied Physics, Aalto University, 00076 Aalto, Finland

[5]Department of Physics, University of South Florida, Tampa, Florida 33620, United States

[6]INSTM Research Unit, University of Padova, Padova Italy

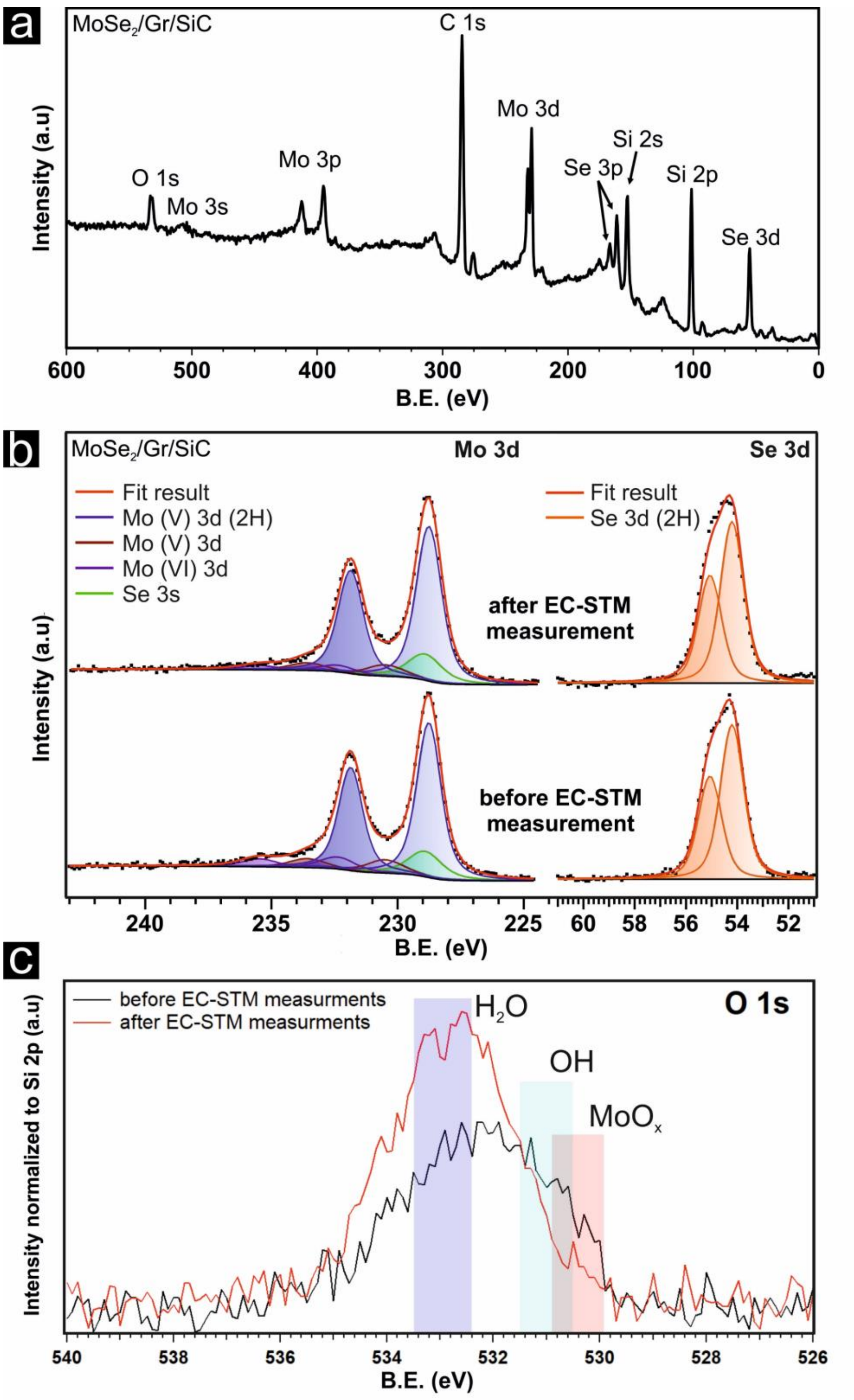

Figure S1: (a) XPS survey spectrum of $MoSe_2$/Gr/SiC sample in prior to in situ EC-STM measurements. (b) Mo 3d and Se 3d high-resolution photoemission spectra of $MoSe_2$/Gr/SiC before and after EC-STM measurements. (c) XPS high-resolution core-level O 1s spectra before and after EC-STM measurements of $MoSe_2$/Gr/SiC. According to the literature, the binding energy of H_2O and

OH are located in the range from 532.4 eV to 533.3 eV and from 530.5 eV and 531.5 eV, respectively.[1,2] Therefore, considering that the sample has been taken out from the electrolyte solution after EC-STM measurements, we observe an increased spectral intensity in the BE position typical of H_2O adsorption. On the other hand, a clear decrease of the intensity in the BE region typical of Mo-oxides is observed in accordance to Mo 3d deconvolution (see Table S1).

Table S1.

	**Mo : Se**	**2H Mo (at. %)**	**MoO_3 (at. %)**	**MoO_x (at. %)**	**2H Se (at. %)**
$MoSe_2$/Gr/SiC as prepared	1.24 : 2	80.7	9.1	10.2	100
$MoSe_2$/Gr/SiC after EC-STM measurement	1.17 : 2	85.7	5.2	9.1	100

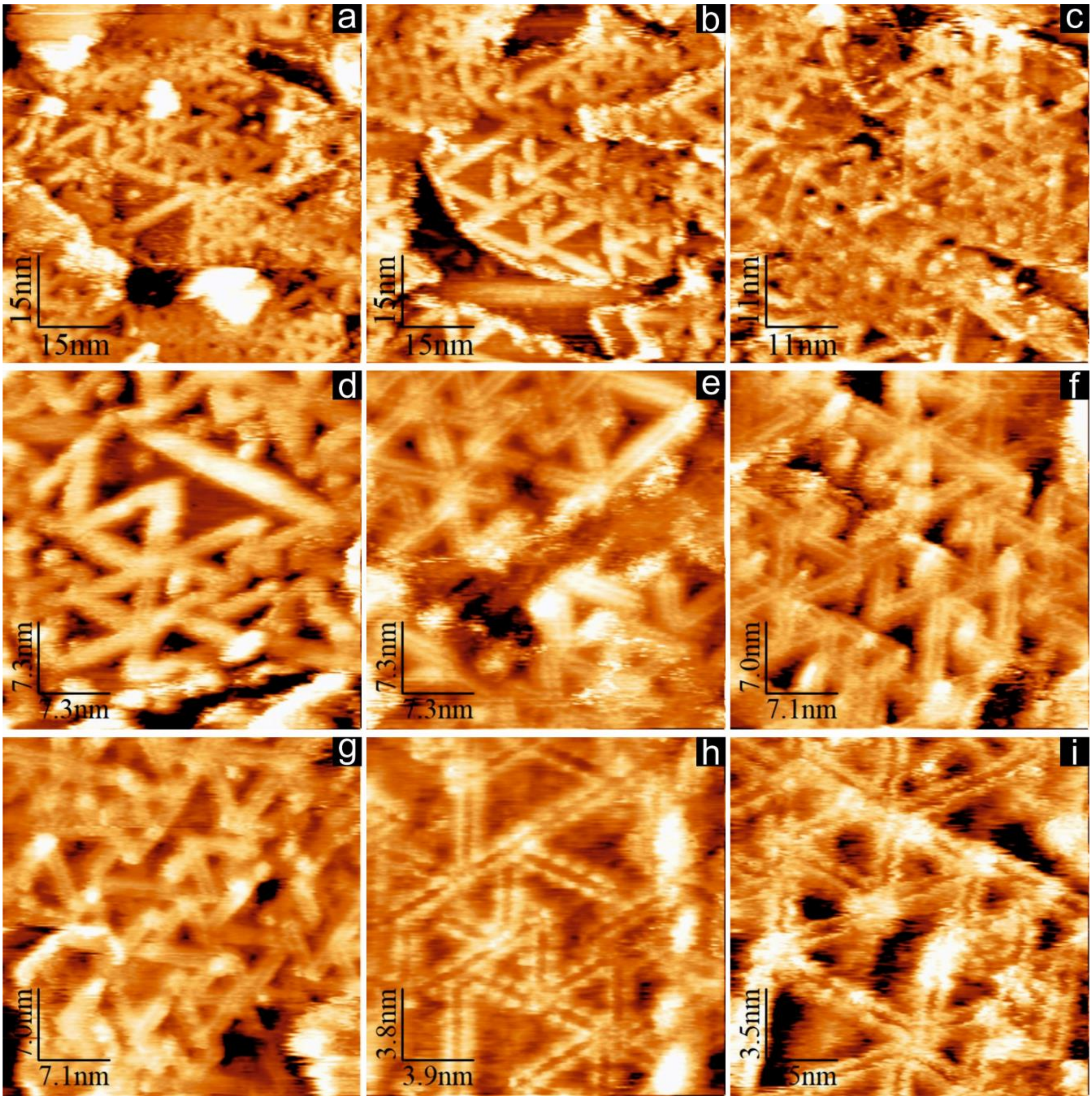

Figure S2: Typical topographic EC-STM images of $MoSe_2$/Gr/SiC (0001)

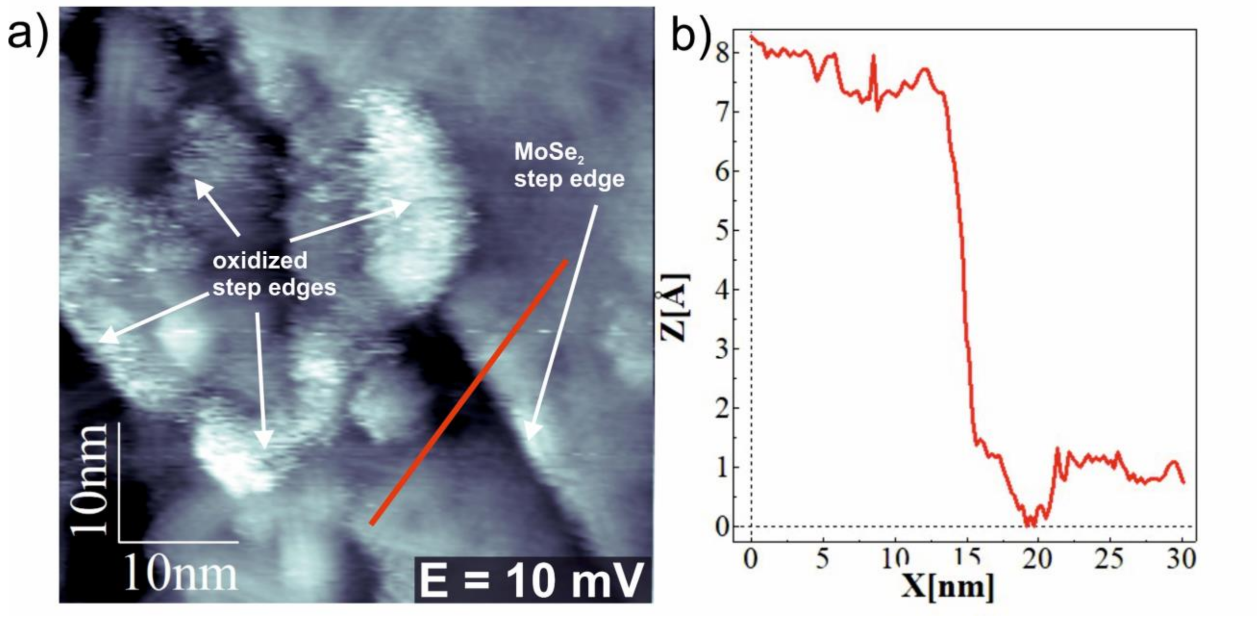

Figure S3: (a) Large scale *in situ* EC-STM image of $MoSe_2$ showing some pristine and oxidized step edges recorded at 10 mV vs RHE, I_T = 1.84 nA, U_B = 89 mV. (b) Height profile measured along the red line in (a).

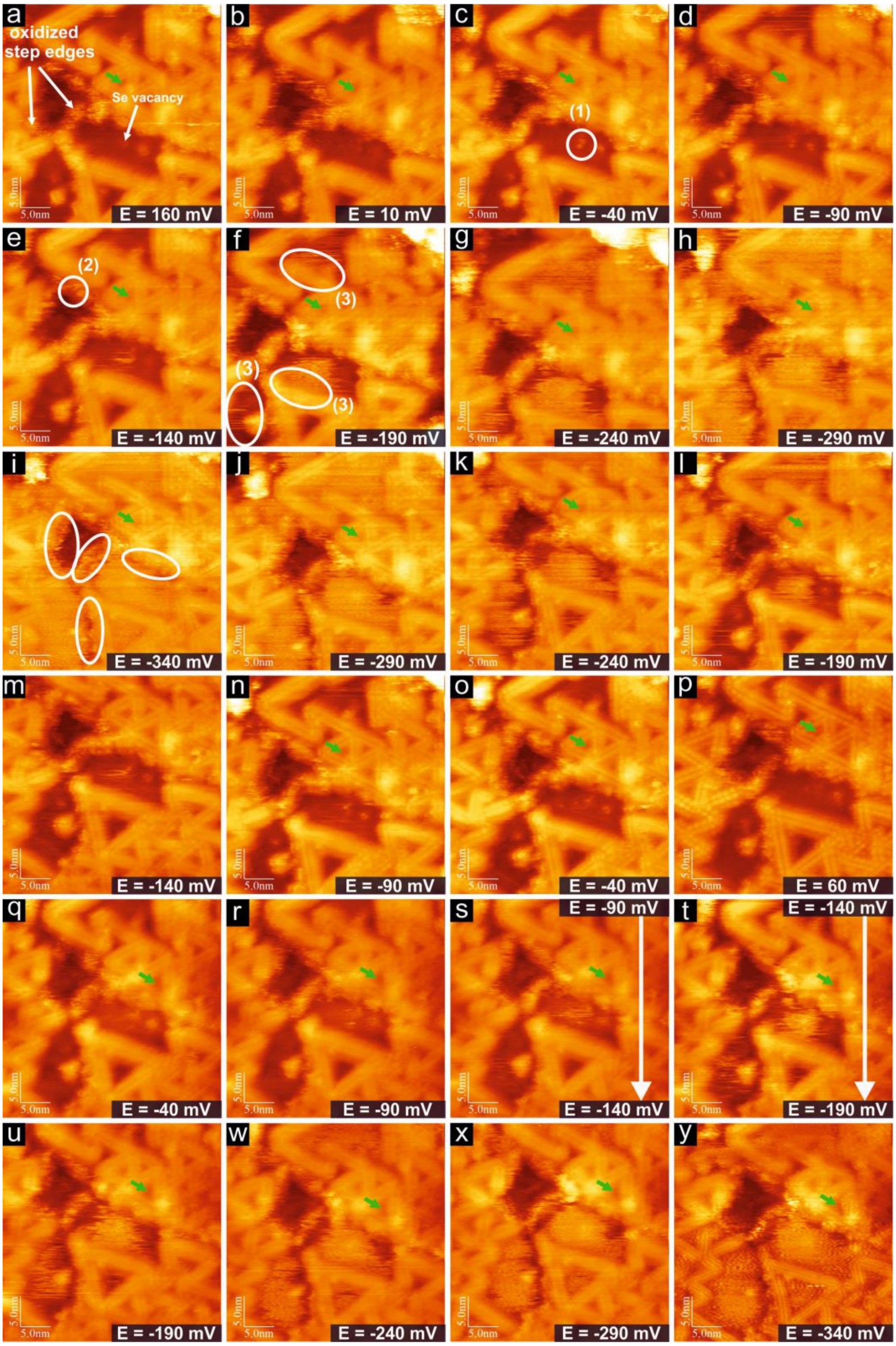

Figure S4: Topographic EC-STM images of $MoSe_2$/Gr/SiC (0001) taken at different E vs RHE: E = 160 mV (a), 10 mV (b), -40 mV (c), -90 mV (d), -140 mV (e), -190 mV (f), -240 mV (g), -290 mV (h), -340 mV (i), -290 mV (j), -240 mV (k), -190 mV (l), -140 mV (m), -90 mV(n), -40 mV (o), 60 mV (p), -40 mV (q), -90 mV(r), from -90 to -140 mV (s), from -140 mV to -190 (t), -190 mV (u), -240 mV (w), -290 mV (x), -340 (mV). Tunnelling conditions: I_T = 1.84 nA, U_B = 89 mV. The green arrow always indicates the same position on the surface.

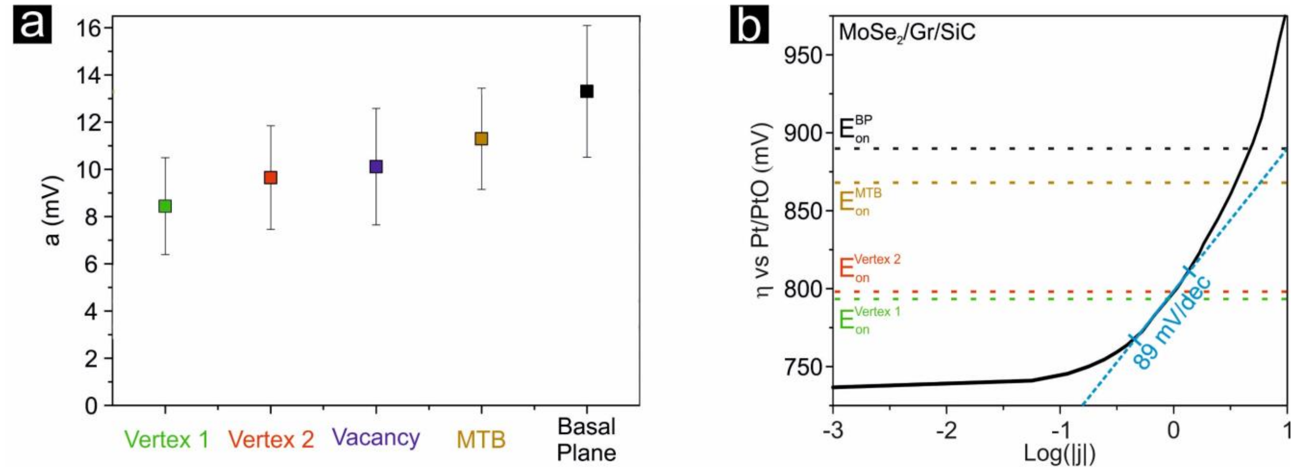

Figure S5: (a) Attenuation factor *a* of the sites highlighted in Figure 2; (b) Tafel plot of the $MoSe_2$/Gr/SiC thin film recorded in Ar-saturated 0.1 M $HClO_4$, scan rate: 20 mVs^{-1}.

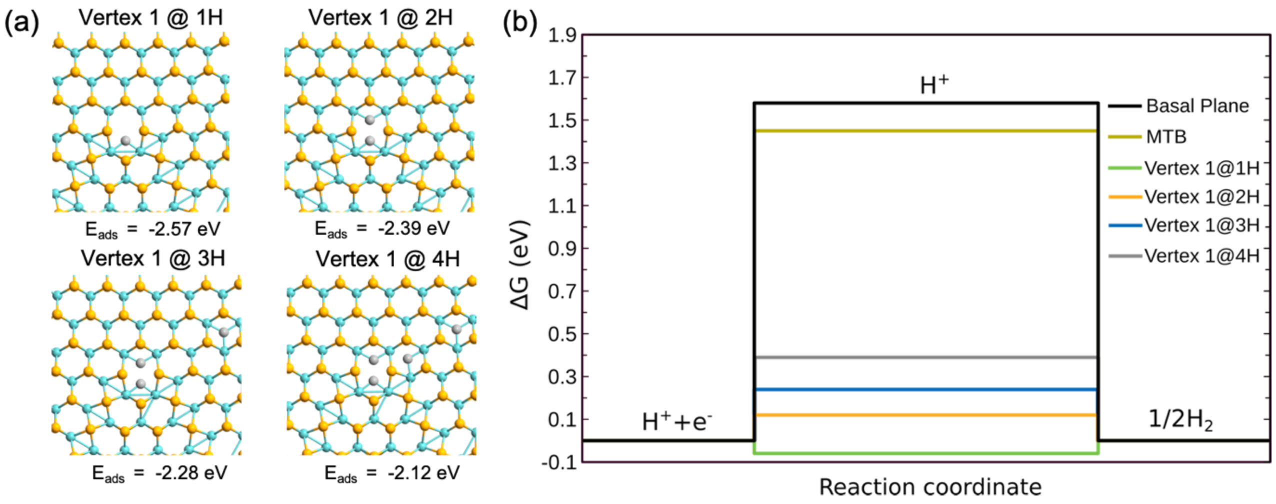

Figure S6: (a) Atomic structures of $MoSe_2$ monolayer with 1-4 H atoms adsorbed on vertex 1. (b) Free energy profiles of HER on pristine $MoSe_2$ monolayer and that with vertex 1 with different concentration of H atoms.

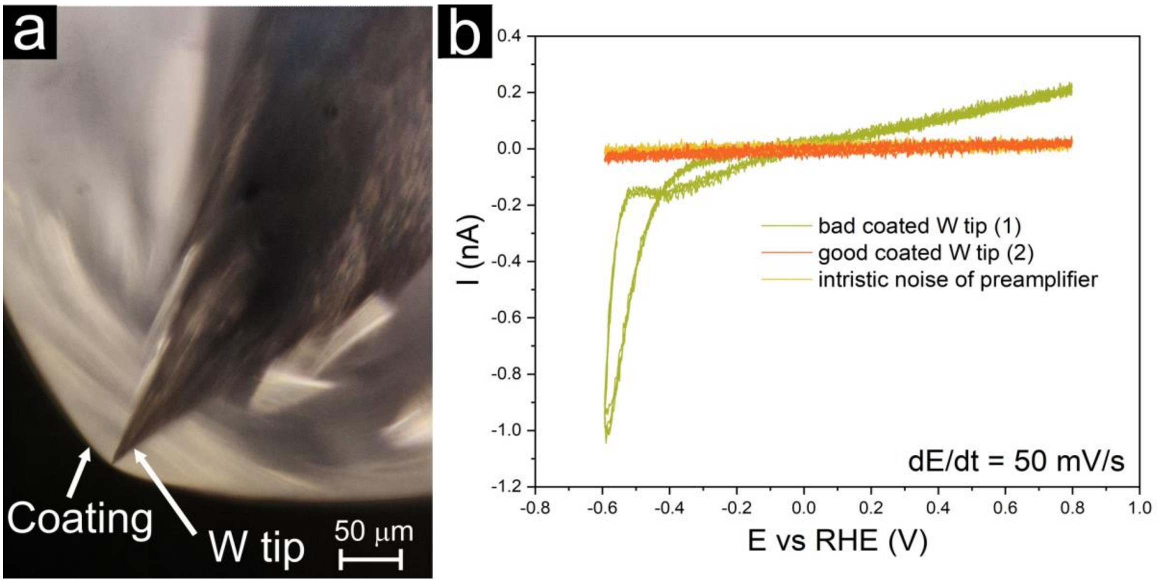

Figure S7: Typical optical microscope image of a coated W tip (a); CVs of a nicely and badly coated W tip recorded in Ar saturated 0.1 M $HClO_4$ and intrinsic noise of STM preamplifier(b).

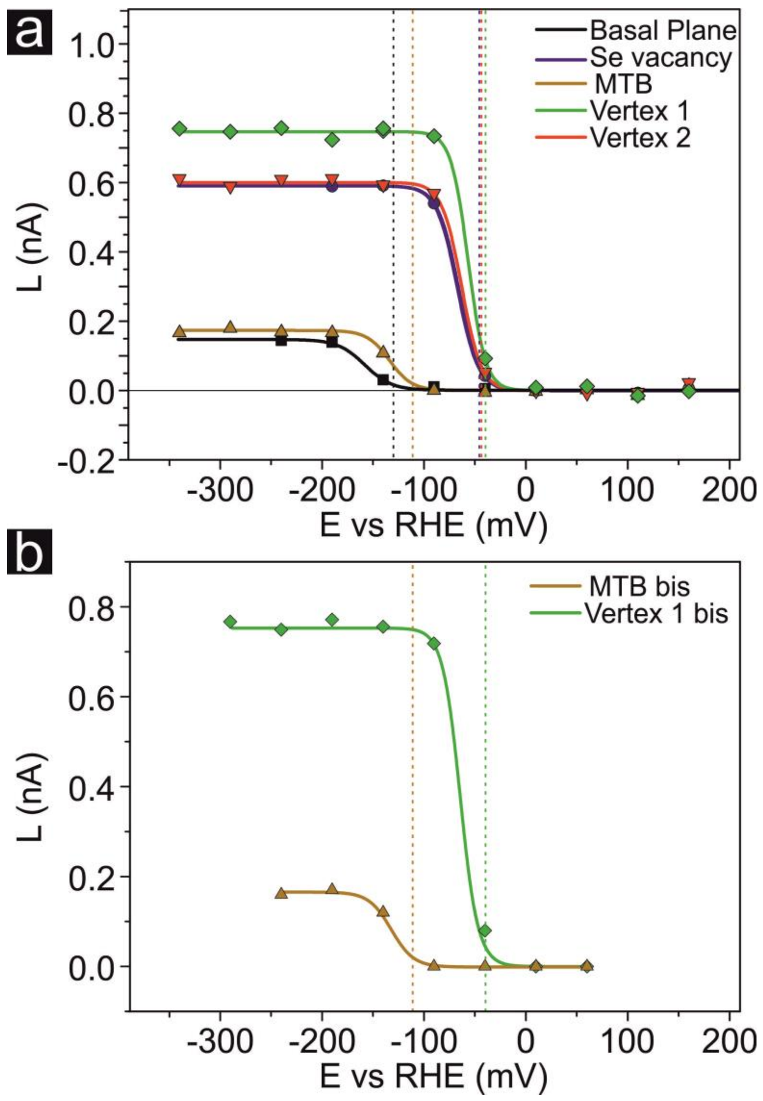

Figure S8: (a) Fit results of the faradaic-cr (L) as a function of the EC potential extracted from the colored rectangles in Figure 2a and (b) additional MTB and Vertex 1 characterizations of the same sites during a different potentiodynamic scan.

Supporting Note 1: Movie description

In-situ potentiodynamic EC-STM experiment which is presented as a movie shows a direct correlation of the changes observed on the surface by the STM as a function of the applied electrode potential in anodic and cathodic direction to the cyclic voltammetry curve. The STM movie is made up by 88 STM images (not filtered) taken one after each other. The time needed to obtain one image and start the next one was around 30s (including manual drift correction during measurement), this means that the observed changes on the surface in the potentiodynamic STM measurement were continuously recorded in approximately 43 min. The STM images were converted into the movie with WSxM software. The movie is exported with the speed of 12fps (frame per second), which with 85 frames gives a total time of 14.5s. The current density and electrode potential at which each STM image is recorded is situated below and on the right side of the 3D rendered STM image. Furthermore, the blue dot placed on the red cyclic voltammetry shows the actual value of the electrode potential. All STM image were recorded using constant tunnelling conditions (I_T = 1.84 nA, U_B = 89 mV). The potentiodynamic STM measurement presented in the movie starts at 160 mV vs RHE. From the starting point the electrode potential was gradually changed in cathodic direction. When the potential reached -340 mV vs RHE the potential sweep was reversed into anodic direction and STM images were continuously recorded till 60 mV vs RHE, when the electrode potential was again changed toward cathodic direction. Then the potential was systematically decreased down to -340 mV vs RHE. During this whole experiment one and a half cycle was covered showing the activation of the hydrogen evolution at different site of the $MoSe_2$.

Supporting Note 2: cr-EC-STM analysis

The current roughness (cr) measures the tunneling current deviation from the set value due to the fluctuation of the tunneling junction resistance produced locally by the ongoing EC reaction. The perturbation can be quantified by the raw-cr (l_0) defined as:

$$l_0 = \sqrt{\frac{\sum_{\mathrm{i,j}}[I(\mathrm{x_i},\mathrm{y_i}) - I_{\mathrm{SET}}]^2}{\mathrm{N}}} \tag{S1}$$

where $I(x_i,y_j)$ are the tunneling current values at the point of (x_i,y_j) coordinates, I_{SET} is the current in the feedback loop, and N is the total number of involved points (i.e. the pixels of the image).

This quantity can be easily calculated on any region of a constant current-mode EC-STM image; thus, any site of interest can be analyzed by choosing a sub-area that encloses it and applying Eq. S1. However, areas with different sizes close to the same site will return different results since the corresponding l_0 values would reflect the different statistical weight that the (x_i,y_j) points of the specific site have compared to the whole investigated area. Therefore, to be independent of the actual size of the chosen area, a size normalization is to be included in Eq S1, defining a size normalized-cr (L_0) as it follows:

$$L_0 = \sqrt{\frac{\sum_{\mathrm{i,j}}[I(\mathrm{x_i},\mathrm{y_i}) - I_{\mathrm{SET}}]^2}{\mathrm{N}} \cdot \frac{A_{SET}}{A_{SITE}}} \tag{S2}$$

where A_{SITE} is the area of *the site* under analysis, and A_{SET} is the *whole area* that was selected for the analysis.

In the presence of electrochemical reactions, the L_0 versus E plots typically show a sigmoidal profile:

$$L_0(\mathrm{E}) = \frac{\Delta L_0}{1 + e^{\frac{E-E_{inf}}{a}}} + L_0^{off} \tag{S3}$$

where E_{inf} i^s the electrochemical potential at the inflection point, ΔL_0 is the amplitude, a is the jump attenuation factor, and L_0^{off} is the background value due to the intrinsic features of the site (i.e., topographic contribution and systematic noise).

When the reaction is off, L_0 has a relatively constant value equal to L_0^{off}. However, when the potential is swept across a potential region where a faradaic reaction is active, the value L_0 progressively increases to a quantity ΔL_0, which represents the contribution added by the faradaic process to the tunneling current. The jump between the on/off conditions can be localized on the electrochemical potential scale through the E_{inf} value. It must be noted that the existence of a plateau at high overpotential has mainly an instrumental origin. The HER onset potential (E_{on}) can be determined by intercept with the x-axis of the tangent to the sigmoid passing through its inflection point.

It is possible to obtain a first estimation of the electroactivity of a specific site by the analysis of the L_0 curves. The same tunneling parameters (bias and tunneling current I_{SET}) must be kept constant

through a potentiodynamic experiment. However, the absolute value of L_0 will depend also on several experimental factors such as acquisition parameters (feedback parameters in the tunneling current circuit) and local apparent corrugation (due to experimental morphological and electronic effects). To eliminate these extrinsic variations of L_0, which however are the same for a certain potentiodynamic series, the value of L_0 before the onset of the catalytic activity, L_0^{off} can be used as a baseline, and the multiplicative constant K as a normalization factor:

$$K = e^{\left|\frac{I_{SET}-I_A}{I_{SET}}\right|} \qquad \text{(S4)}$$

This last parameter considers that on highly corrugated surfaces, the value of I_{SET} and I average (I_A) can be significantly different, especially for atomically resolved images on highly corrugated surfaces. Therefore, taking into account the Eqs S3 and S4, another cr value (L) can be defined:

$$L(E) = \frac{(L_0(E) - L_0^{off})}{K} \qquad \text{(S5)}$$

The value L describes the size- normalized local deviation of the recorded tunneling current from the set value due to the electrochemical contribution only, so it is named faradaic-cr value.

The L(E) versus E plot values show sigmoidal profiles similar to Eq. S3:

$$L(E) = \frac{\Delta L}{1 + e^{\frac{E-E_{inf}}{a}}} \qquad \text{(S6)}$$

where ΔL is the normalized faradaic process contribution.

Supporting Note 3: TOF measurement per vertex sites

The turnover frequency (TOF) is calculated using the macroscopic current density j and the active site density ρ_{SITE}

$$TOF(E) = \frac{Total\ number\ of\ H_2\ per\ second}{Total\ number\ of\ active\ site} = \frac{j(E)}{2 \cdot q \cdot \rho_{SITE}} \qquad \text{(S7)}$$

where q is the elementary charge ($-1.6 \cdot 10^{-19}$ C), and 2 accounts for 2 H atoms per H_2 molecule.
Based on the performed cr-analysis (Figure 3b), it was decided to neglect the differences in the activity between the possible different vertex types. Hence the ρ_{SITE} value per the vertex site was statistically estimated from the topographic images in Figures 1a and S2 with a cumulative vertices density of approximately $3.6 \cdot 10^{12}$ sites·cm^{-2}. An individual H_2 evolution site for each vertex was evaluated in the count of the active site, considering two possible Volmer-Tafel mechanism cases: the adsorption of two H atoms, either both on the Vertex 1 site or one on it and one on an adjacent basal plane site (see Figure S6a). At last, estimating the coverage of the MTB area (areas with triangle pattern) of about 80%, the used ρ_{SITE} value has been $2.9 \cdot 10^{12}$ sites·cm^{-2}.
A correction factor of 0.8 V was considered for the conversion of the potential of the Pt/PtO reference electrode to the RHE.[3]

The literature values reported for comparing TOF values in Figure 4a were calculated in accordance with Eq S7.[4–8] In detail: the surface density of the active sites was estimated by taking into consideration the number of Mo atoms of the basal plane calculated form crystallographic data in case of the basal plane of (2H or 1T) MoS_2 and $MoSe_2$. [4–6,8] In the case of defective materials, activated by the presence of vacancies or step edges, the actual number of active sites was determined by XPS[4] measurements or by STM.[7]

Supplementary Note 4: EC-STM tip

Figure S7 shows a typical optical microscope image of a well-coated tip, in which the glue coating is visible up to the apex of the W tip. Further details on the quality of the tips can be deduced from the CV measurements. The typical CV response of a well coated W tip in a 0.1 M $HClO_4$ Ar$^-$ sat. el. is shown in Figure S7b. The faradaic contribution is close to the detection limit of the STM preamplifier (see for the comparison the I-V curve of the intrinsic noise of preamplifier). Consequently, the faradaic current component flowing through the tip during STM imaging is far below the value of the real tunneling current and it is independent from the applied potential.

Supporting Note 5: DFT calculation

DFT calculations were carried out using the plane-wave basis set with the projector-augmented wave description of the core regions, as implemented in the VASP code[9,10]. The calculations were performed using the generalized gradient approximation with PBE exchange-correlation functional[11]. The cutoff energy of plane waves was set to 450 eV. To model slabs, a vacuum space of around 20 Å was considered between the monolayers in the confinement directions. The force tolerance was set to 0.01 eV for geometry optimizations. The Brillouin zone of the supercells was sampled using gamma point approximation. The van der Waals interactions were taken into account as proposed by Grimme within the DFT-D2 method[12]. A 6×6 supercell was used for simulations of hydrogen adsorption on the monolayer with vacancy and MTBs. The structural models for vertices include non-periodic flakes with a maximum number of 312 atoms.

The adsorption energy (E_{ads}) of the adsorbate on the surface is calculated as:

$$\mathrm{E_{ads}} = \mathrm{E_{slab+adsorbate}} - \mathrm{E_{slab}} - \mathrm{E_{adsorbate}} \quad \text{(S8)}$$

where the $E_{\mathrm{slab+adsorbate}}$ represents the total energy of the slab with adsorbate, E_{slab} indicates the energy of the isolated slab, and $E_{\mathrm{adsorbate}}$ stands for the energy of the isolated adsorbate.

The HER performance of the systems can be characterized by the Gibbs free reaction energy ΔG, according to the following equation[13]:

$$\Delta G_H = \Delta E_{ads} + \Delta E_{ZPE} - T\Delta S_H \quad \text{(S9)}$$

ΔE_{ads} represents the change in the internal energy, ΔE_{ZPE} is the change in the vibrational zero-point energy, *and* ΔS indicates the difference in entropy. Because the catalyst's contributions to ΔE_{ZPE} and $T\Delta S_H$ are so negligible, they can be disregarded. ΔE_{ZPE} value can range from -0.01 to 0.04 eV, as shown previously;[14] while the entropy of atomic hydrogen (ΔS_H) atom can be estimated to $-0.5 \cdot \Delta S_H^0$, where ΔS_H^0 is the entropy of a gas phase H_2 molecule. The value of TS_H^0 is approximated to 0.4 eV.[13] Finally, the term $\Delta E_{ZPE} - T\Delta S_H$ can be estimated to be +0.24 eV at room temperature.[13,15,16] We consider this value to be representative for all the calculations in this work. Therefore, Eq S9 can be simplified to $\Delta G_H = \Delta E_H + 0.24$ eV.

REFERENCES

(1) Yamamoto, S.; Bluhm, H.; Andersson, K.; Ketteler, G.; Ogasawara, H.; Salmeron, M.; Nilsson, A. In Situ X-Ray Photoelectron Spectroscopy Studies of Water on Metals and Oxides at Ambient Conditions. *J. Phys. Condens. Matter* **2008**, *20* (18). https://doi.org/10.1088/0953-8984/20/18/184025.

(2) Yamamoto, S.; Kendelewicz, T.; Newberg, J. T.; Ketteler, G.; Starr, D. E.; Mysak, E. R.; Andersson, K. J.; Ogasawara, H.; Bluhm, H.; Salmeron, M.; Brown, G. E.; Nilsson, A. Water Adsorption on α-Fe2O3 (0001) at near Ambient Conditions. *J. Phys. Chem. C* **2010**, *114* (5), 2256–2266. https://doi.org/10.1021/jp909876t.

(3) Filoni, C.; Wandelt, K.; Marfori, L.; Leone, M.; Duò, L.; Ciccacci, F.; Bussetti, G. A Combined EC-STM and EC-AFM Investigation of the Sulfate Adsorption on a Cu(111) Electrode Surface up to the Anodic Corrosion Potential. *Appl. Surf. Sci.* **2022**, No. 111, 155542. https://doi.org/10.1016/j.apsusc.2022.155542.

(4) Li, H.; Tsai, C.; Koh, A. L.; Cai, L.; Contryman, A. W.; Fragapane, A. H.; Zhao, J.; Han, H. S.; Manoharan, H. C.; Abild-Pedersen, F.; Nørskov, J. K.; Zheng, X. Activating and Optimizing MoS2 Basal Planes for Hydrogen Evolution through the Formation of Strained Sulphur Vacancies. *Nat. Mater.* **2016**, *15* (1), 48–53. https://doi.org/10.1038/nmat4465.

(5) Xia, B.; Wang, T.; Jiang, X.; Zhang, T.; Li, J.; Xiao, W.; Xi, P.; Gao, D.; Xue, D.; Ding, J. Ar2+ Beam Irradiation-Induced Multivancancies in MoSe2 Nanosheet for Enhanced Electrochemical Hydrogen Evolution. *ACS Energy Lett.* **2018**, *3* (9), 2167–2172. https://doi.org/10.1021/acsenergylett.8b01209.

(6) Zhao, G.; Li, P.; Rui, K.; Chen, Y.; Dou, S. X.; Sun, W. CoSe2/MoSe2 Heterostructures with Enriched Water Adsorption/Dissociation Sites towards Enhanced Alkaline Hydrogen Evolution Reaction. *Chem. - A Eur. J.* **2018**, *24* (43), 11158–11165. https://doi.org/10.1002/chem.201801693.

(7) Voiry, D.; Salehi, M.; Silva, R.; Fujita, T.; Chen, M.; Asefa, T.; Shenoy, V. B.; Eda, G.; Chhowalla, M. Conducting MoS2 Nanosheets as Catalysts for Hydrogen Evolution Reaction. *Nano Lett.* **2013**, *13* (12), 6222–6227. https://doi.org/10.1021/nl403661s.

(8) Benck, J. D.; Hellstern, T. R.; Kibsgaard, J.; Chakthranont, P.; Jaramillo, T. F. Catalyzing the Hydrogen Evolution Reaction (HER) with Molybdenum Sulfide Nanomaterials. *ACS Catal.* **2014**, *4* (11), 3957–3971. https://doi.org/10.1021/cs500923c.

(9) Kresse, G.; Furthmüller, J. Efficiency of Ab-Initio Total Energy Calculations for Metals and Semiconductors Using a Plane-Wave Basis Set. *Comput. Mater. Sci.* **1996**, *6* (1), 15–50. https://doi.org/10.1016/0927-0256(96)00008-0.

(10) Kresse, G.; Furthmüller, J. Efficient Iterative Schemes for Ab Initio Total-Energy Calculations Using a Plane-Wave Basis Set. *Phys. Rev. B - Condens. Matter Mater. Phys.* **1996**, *54* (16), 11169–11186. https://doi.org/10.1103/PhysRevB.54.11169.

(11) Perdew, J. P.; Burke, K.; Ernzerhof, M. Generalized Gradient Approximation Made Simple. *Phys. Rev. Lett.* **1996**, *77* (18), 3865–3868. https://doi.org/10.1103/PhysRevLett.77.3865.

(12) Grimme, S. Semiempirical GGA-Type Density Functional Constructed with a Long-Range Dispersion Correction. *J. Comput. Chem.* **2006**, *27* (15), 1787–1799. https://doi.org/10.1002/jcc.20495.

(13) Nørskov, J. K.; Bligaard, T.; Logadottir, A.; Kitchin, J. R.; Chen, J. G.; Pandelov, S.; Stimming, U. Trends in the Exchange Current for Hydrogen Evolution. *J. Electrochem. Soc.* **2005**, *152* (3), J23. https://doi.org/10.1149/1.1856988.

(14) Tsai, C.; Abild-Pedersen, F.; Nørskov, J. K. Tuning the MoS2 Edge-Site Activity for Hydrogen Evolution via Support Interactions. *Nano Lett.* **2014**, *14* (3), 1381–1387. https://doi.org/10.1021/nl404444k.

(15) Skúlason, E.; Tripkovic, V.; Björketun, M. E.; Gudmundsdóttir, S.; Karlberg, G.; Rossmeisl, J.; Bligaard, T.; Jónsson, H.; Nørskov, J. K. Modeling the Electrochemical Hydrogen Oxidation and Evolution Reactions on the Basis of Density Functional Theory Calculations. *J. Phys. Chem. C* **2010**, *114* (42), 18182–18197. https://doi.org/10.1021/jp1048887.

(16) Greeley, J.; Mavrikakis, M. Surface and Subsurface Hydrogen: Adsorption Properties on Transition Metals and near-Surface Alloys. *J. Phys. Chem. B* **2005**, *109* (8), 3460–3471. https://doi.org/10.1021/jp046540q.
